# Recent developments of acupuncture in Australia and the way forward

**DOI:** 10.1186/1749-8546-4-7

**Published:** 2009-04-29

**Authors:** Charlie Changli Xue, Anthony Lin Zhang, Angela Weihong Yang, Claire Shuiqing Zhang, David Frederick Story

**Affiliations:** 1 World Health Organisation Collaborating Centre for Traditional Medicine, Discipline of Chinese Medicine, School of Health Sciences, RMIT University, Bundoora, Victoria 3083, Australia

## Abstract

Almost one in ten Australians has received acupuncture treatment by acupuncturists and/or medical doctors in private clinics. The majority of Australian health insurance funds offer rebates for acupuncture. Statutory regulations for acupuncture have been implemented in the State of Victoria, Australia. Six acupuncture degree courses have been approved by the Chinese Medicine Registration Board of Victoria and/or accredited by the Australian Acupuncture and Chinese Medicine Association. Furthermore, a number of clinical trials of acupuncture on allergic rhinitis, pain and women's health were carried out in Australia. Recent developments of acupuncture in Australia indicate that through adequate and appropriate evaluation, acupuncture begins to integrate into mainstream health care in Australia.

## Background

The history of acupuncture in Australia can be traced back to the 1850s when the first Chinese immigrants arrived and worked in the gold fields of Australia [1]. Acupuncture is now considered by the general public as one of the most popular treatments of complementary and alternative medicine (CAM) [2].

There have been four developmental stages of acupuncture in Australia. (1) Self-management stage (1850s–1960s): Acupuncture was a form of unregulated health care. (2) Professional development stage (1970s–1980s): Acupuncture associations were established to promote the acupuncture profession and facilitate clinical practices. (3) Standard-setting stage (1990s): Universities and private colleges started offering acupuncture training. Acupuncture became an established modality of CAM in Australia [3]. (4) Regulation stage (2000 onwards): The practice of acupuncture is subject to mandatory registration in the State of Victoria, Australia as stipulated by the Chinese Medicine Registration Act 2000 [4] which was superseded by the Health Professions Registration Act 2005 [5].

Here, we highlight the recent developments of acupuncture in terms of clinical practices, education, research and regulations in Australia and illustrate how acupuncture is being integrated into mainstream health care in Australia.

## Acupuncture users and practitioners in Australia

In 2005, a national survey on 1,067 representative adults in Australia on the use of CAM revealed that nearly one in ten (9.2%) Australians used acupuncture service over a 12-month period [2]. Australians made over ten million visits to acupuncturists every year. The study also concluded that acupuncture users were more likely to be born in Australia, having completed tertiary education, covered by private health insurance and living in the states of New South Wales, Victoria and Queensland [6].

Acupuncture can be provided by both acupuncturists and general medical practitioners with training in acupuncture. A 2004 survey on 636 medical practitioners showed that acupuncture was considered as one of the three most popular forms of CAM used by medical practitioners themselves [7]. Nearly one in five (18%) general medical practitioners practiced acupuncture as part of their primary care and 76% of medical doctors referred their patients to acupuncturists at least once a month [7]. Over 70% of obstetricians and midwives considered acupuncture to be beneficial and safe to use during pregnancy according to another study on 220 obstetricians and midwives in Australia [8].

Over the past three decades, a number of Chinese medicine and acupuncture associations have been established in Australia, among which the Australian Acupuncture and Chinese Medicine Association (AACMA) is the largest one in Australia with over 1,400 members [9].

## Acupuncture education

In Australia, private acupuncture colleges were first established in Sydney, Brisbane and Melbourne in the 1970s [9]. The Victoria University of Technology and the Royal Melbourne Institute of Technology (RMIT University) were among the first to offer degree courses in acupuncture and Chinese herbal medicine.

Twenty universities and colleges in Australia offer Chinese medicine courses at various levels which may also include acupuncture. Some of the acupuncture programs have been approved by the Chinese Medicine Registration Board of Victoria (CMRB) or recognised by the AACMA (Table 1). Moreover, the RMIT University, University of Technology Sydney and University of Western Sydney also offer research programs for acupuncture stream at postgraduate level. The State of Victoria has issued the Guidelines for the Approval of Courses of Study in Chinese Medicine as a Qualification for Registration [10].

**Table 1:** List of acupuncture degree courses available in Australia

**Course**	**Institution**	**Approving/Recognising bodies**
Bachelor of Health Science (acupuncture): 4-year undergraduate course	Endeavour College of Natural Health (previously Australian College of Natural Medicine), Queensland and Victoria	CMRB*, AACMA
Bachelor of Applied Science (Chinese medicine/human biology): 5-year undergraduate course	RMIT University, Victoria	CMRB, AACMA
Master of Applied Science (acupuncture): 3-year postgraduate course	RMIT University, Victoria	CMRB
Bachelor of Health Science (Chinese medicine): 4-year undergraduate course	Southern School of Natural Therapies, Victoria	CMRB*, AACMA*
Bachelor of Health Science in traditional Chinese medicine: 4-year undergraduate course	University of Technology Sydney, New South Wales	AACMA
Bachelor of Applied Science (traditional Chinese medicine): 4-year undergraduate course	University of Western Sydney, New South Wales	AACMA

Note:

CMRB: Chinese Medicine Registration Board of Victoria

AACMA: Australian Acupuncture and Chinese Medicine Association

*Provisional approval/recognition

A comparative study on the curricula and teaching quality between the RMIT University and Beijing University of Chinese Medicine was recently conducted [11]. While the curricula and educational objectives of the two programs were similar, differences existed in areas such as teaching classical Chinese medicine texts and clinical training in Chinese medicine hospitals. A survey on 228 registered Chinese medicine practitioners in Victoria was conducted in 2005 [12]. Its results showed that technical capabilities (acupuncture in particular) were considered as the most important in clinical practice, whereas research was considered the least important.

## Acupuncture regulation in Australia

The Australian Government issued the Standards of Practice for Acupuncture – Health (Infectious Diseases) Regulations 1990 [13]. In 2000, the State of Victoria implemented the Chinese Medicine Registration Act [4] for the purpose of registration of Chinese medicine practitioners. This Act was subsequently replaced by the Health Professions Registration Act 2005 [5]. By April 2009, the CMRB has registered 987 acupuncturists who met the requirements of the Act [14].

## Health insurance rebates for acupuncture

At present, Australian national health insurance (i.e. Medicare) only covers the cost of acupuncture provided by registered medical practitioners. Between 2005 and 2006, the number of medical acupuncture visits rebated by Medicare was estimated to be 607,349 [15], representing less than 10% of the total estimated acupuncture visits per year [7]. Since the 1990s, most of the private health insurance companies in Australia, such as Medibank Private and Medical Benefits Fund of Australia, have been providing rebates for acupuncture.

## Research on acupuncture efficacy and safety

The RMIT Chinese Medicine Research Group (RCMRG) conducted randomised controlled trials (RCTs) on acupuncture as a treatment for allergic rhinitis, headache, migraine and chronic pain. An RCT involving 30 subjects [16] with seasonal allergic rhinitis showed a significant improvement after acupuncture treatment for nasal and non-nasal symptoms. Another RCT [17] involving persistent allergic rhinitis subjects (*n *= 80) showed that the symptom scores in the acupuncture group decreased significantly. These two RCTs suggest that acupuncture may be an effective and safe method to treat seasonal and persistent allergic rhinitis.

An RCT (*n *= 40) conducted by the RCMRG examined the efficacy of electroacupuncture for tension-type headache [18], concluding that electroacupuncture was effective for short-term symptomatic relief. In addition, a pilot RCT in a hospital in Victoria demonstrated that electroacupuncture had beneficial effects on short-term reduction of Opioid-like medication in subjects with chronic non-malignant pain [19].

An RCT conducted at the University of Adelaide in South Australia involving 593 women with nausea and vomiting during early pregnancy suggested that acupuncture was generally safe for women of early pregnancy and that women receiving acupuncture had less nausea (*P *< 0.01) throughout the trial and less dry retching (*P *< 0.01) the second week onwards [20]. Another RCT on 228 patients found positive effects of acupuncture on clinical pregnancy rates for women undergoing embryo transfer [21].

Researchers in Australia have also made considerable contributions to the Cochrane Database of Systematic Reviews in the investigation on acupuncture to treat depression [22], induction of labour [23], lateral elbow pain [24], shoulder pain [25] and fibromyalgia [26].

Laser acupuncture is an alternative method to traditional acupuncture. Findings from an RCT involving 30 subjects with mild to moderate depression showed that the laser acupuncture group had significantly lower depression scores than did the inactive laser acupuncture group [27].

Most acupuncture clinical trials conducted in Australia are registered with the Australian Therapeutic Goods Administration and the Australian New Zealand Clinical Trial Registry. Following the introduction of the Consolidated Standards of Reporting Trials (CONSORT Statement) in 1996 [28] and its revised version in 2001 [29], clinical trials in Australia have been reported in accordance with this international standard.

Challenges in acupuncture clinical trials remain, especially in effective design and control group such as sham [30]. Researchers have identified some strategies to maintain the credibility of sham acupuncture as a control in clinical trials [31].

Against the backdrop of increasing Chinese medicine research in Australia, the Australian Journal of Acupuncture and Chinese Medicine was launched in 2006 as the first peer-reviewed journal on Chinese medicine in Australia.

## Conclusion

Recent developments of acupuncture in Australia indicate that through adequate and appropriate evaluation, acupuncture is being integrated into mainstream health care in Australia.

## Competing interests

The authors declare that they have no competing interests.

## Authors' contributions

CX, AZ, AY and DS conceived the ideas of this article. CX, AZ, AY and SZ collected and compiled the data, and drafted the manuscript. CX, AZ, AY, SZ and DS interpreted the data and revised the manuscript. All authors read and approved the final version of the manuscript.
